# The Atherogenic Role of Circulating Modified Lipids in Atherosclerosis

**DOI:** 10.3390/ijms20143561

**Published:** 2019-07-20

**Authors:** Volha I. Summerhill, Andrey V. Grechko, Shaw-Fang Yet, Igor A. Sobenin, Alexander N. Orekhov

**Affiliations:** 1 Institute for Atherosclerosis Research, Skolkovo Innovative Center, Moscow 121609, Russia; 2 Federal Research and Clinical Center of Intensive Care Medicine and Rehabilitology, 14-3 Solyanka Street, Moscow 109240, Russia; 3 Institute of Cellular and System Medicine, National Health Research Institutes, 35 Keyan Road, Zhunan Town, Miaoli County 35053, Taiwan; 4 Laboratory of Medical Genetics, National Medical Research Center of Cardiology, 15A 3-rd Cherepkovskaya Street, Moscow 121552, Russia; 5 Institute of Human Morphology, 3 Tsyurupa Street, Moscow 117418, Russia; 6 Institute of General Pathology and Pathophysiology, 8 Baltiiskaya Street, Moscow 125315, Russia

**Keywords:** atherosclerosis, modified low-density lipoprotein, desialylation, LDL-CIC, trans-sialidase

## Abstract

Lipid accumulation in the arterial wall is a crucial event in the development of atherosclerotic lesions. Circulating low-density lipoprotein (LDL) is the major source of lipids that accumulate in the atherosclerotic plaques. It was discovered that not all LDL is atherogenic. In the blood plasma of atherosclerotic patients, LDL particles are the subject of multiple enzymatic and non-enzymatic modifications that determine their atherogenicity. Desialylation is the primary and the most important atherogenic LDL modification followed by a cascade of other modifications that also increase blood atherogenicity. The enzyme trans-sialidase is responsible for the desialylation of LDL, therefore, its activity plays an important role in atherosclerosis development. Moreover, circulating modified LDL is associated with immune complexes that also have a strong atherogenic potential. Moreover, it was shown that antibodies to modified LDL are also atherogenic. The properties of modified LDL were described, and the strong evidence indicating that it is capable of inducing intracellular accumulation of lipids was presented. The accumulated evidence indicated that the molecular properties of modified LDL, including LDL-containing immune complexes can serve as the prognostic/diagnostic biomarkers and molecular targets for the development of anti-atherosclerotic drugs.

## 1. Introduction

Cardiovascular diseases (CVDs) are responsible for the high mortality and morbidity rates among adults worldwide posing a major socioeconomic burden not only on the health care system of a country but also on the whole national economic growth. The WHO World Heart Day (2017) has declared that the death toll in 2016 due to CVDs was an estimated 17.9 million people representing 31% of all causes of deaths in the world [1]. The global cost of CVD management was $863 billion in 2010 alone, with an expected increase of 22% by 2030 [2,3].

Atherosclerosis is the main underlying cause of life-threatening CVDs. Relying on the population-based observational studies, it was established that the high incidence of atherosclerosis is prevalent among older adults in societies that adopt the Western pattern of diet and lifestyle [4,5,6]. In that regards, various risk factors contributing to the development of atherosclerosis were identified, including tobacco smoking, hypertension, dyslipidemia, hyperglycemia/insulin resistance, overweight/obesity, and genetic predisposition [7]. According to the current understanding, these conditions trigger vascular damage and lipid penetration into the vascular wall. In particular, atherogenic dyslipidemia plays an important role in the development of atherosclerotic lesions. Hypercholesterolemia was reported as the highest attributable risk factor for atherosclerosis and subsequent coronary heart disease in a given population [8]. Moreover, it was shown that persistently elevated levels of LDL were directly associated with the progression from early stage fatty streaks to advanced-stage, lipid-rich lesions [8]. In addition, ethnicity also may determine the incidence, severity, and age/sex distribution of atherosclerosis. A cohort study demonstrated that these parameters were higher among white American older men than in other ethnic groups, even after the adjustment of modifiable risk factors [9].

Furthermore, there is strong evidence that atherosclerosis affects young people and its prevalence and extent increase with age. Numerous studies reported that the subclinical form of atherosclerosis is frequently present in a large population of young adults in association with the presence of atherosclerosis risk factors [10,11,12,13,14]. In fact, the incidence of clinically silent atherosclerotic lesions may reach up to 100% in this cohort of subjects [15]. Young patients exhibit variations in etiologies and risk-factor profiles compared to older patients, resulting in differences in disease progression, prognosis, and treatment. The development of atherosclerotic lesions in young people can be attributed to the assumption that compared to older people, they are more likely to be smokers, male, obese, drug users, and have a positive family history. In particular, cocaine and other illegal drug use have been increasingly linked to accelerated atherosclerosis and acute myocardial infarction in teenagers [16]. Asymptomatic atherosclerosis can have a prolonged latent period lasting for many years, even decades, prior to the first onset of clinical symptoms. In many cases, the acute ischemia of organs is its first clinical manifestation and is often fatal [15].

### Development of Atherosclerotic Lesions: Background

Human atherosclerosis is a complex systemic inflammatory disorder characterized by the interplay between several different moieties, including various lipids, enzymes, endothelial cells, cytokines, and circulating mononuclear cells. Such interactions lead to the thickening of the arterial wall due to lipid accumulation in the intimal layer, endothelial dysfunction, and inflammatory and fibroproliferative responses resulting in vascular proliferation, alterations in the extracellular matrix, and the formation of atherosclerotic plaques. The formation of atherosclerotic lesions takes place at specific arterial regions (branching sites), where low and oscillatory endothelial shear stress occurs [17]. Atherosclerotic plaques develop predominantly in the walls of large and medium-sized arteries causing blood vessel occlusion, as a result of either arterial wall thickening or formation of a thrombus on the surface of unstable plaques [18]. The latter condition is particularly dangerous since it can lead to sudden thromboembolism and death. Atherosclerosis is a life-long condition developing progressively through the constant evolution of lesions in the vascular wall. Histologic changes are increasingly diverse and can vary considerably among individuals. Clinical manifestations of atherosclerosis appear at the later stages, while early atherosclerosis is asymptomatic.

Subendothelial lipid accumulation plays a crucial role in the development of atherosclerotic lesions in the arterial cells. The extra- and intracellular lipid deposition predominantly of cholesterol esters leading to the formation of foam cells in the arterial intima is not only one of the earliest manifestations of atherosclerosis (preclinical atherogenesis) but also the triggering event of the onset of atherosclerotic lesion development [19]. The cells populating atherosclerotic lesions are called foam cells for the foamy appearance of their cytoplasm, which is almost completely loaded with lipid inclusions [20]. The accumulation of foam cells in the arterial intimal cells leads to the formation of the initial lesion and the subsequent fatty streaks that represent early stage lesions in the proatherogenic progression. Moreover, intracellular cholesterol retention is associated with the increase in proliferative activity, as well as the increased synthesis of extracellular matrix components in subendothelial cells [21]. Thus, intracellular lipid accumulation is tightly implicated in the development of all the major manifestations of atherosclerosis at the cellular level.

Therefore, in the study of atherogenesis, the early event of the atherosclerotic process is particularly important. In this regard, the mechanisms of atherogenic modifications of low-density lipoprotein (LDL) particles in the blood of atherosclerotic patients attracted special attention. In this review, we will discuss the molecular properties of circulating modified low-density lipoprotein that trigger atherogenesis, highlighting the importance of its desialylation.

## 2. The Concept of Multiple Modifications of LDL in Atherogenesis

The hypothesis of cholesterol retention in the arterial cells linked to the high levels of total cholesterol in the blood that trigger initiation and further progression of atherosclerosis was proposed by Nikolai Anitschkow over 100 years ago. However, later it was established that lipid deposition in the arterial intima is not associated with the total cholesterol levels but with the high levels of the atherogenic LDL cholesterol [14,22,23]. It was shown that there is a positive correlation between the blood atherogenic properties and plasma ratios of modified lipids, which may be related to the imbalance between the concentrations of modified LDL and high-density lipoprotein (HDL) [21]. Noteworthily, HDL plays a protective role in atherosclerosis [24].

Following the lipid theory of atherosclerosis, LDL is the main transporter of serum cholesterol to the target vascular cells [25], therefore, LDL serum concentration is the main determinant of the intracellular deposition of lipids or lipidosis. Noteworthily, LDL particles are particularly enriched with non-esterified, so-called free cholesterol accounting up to 50% of the particle weight, in comparison with other lipoprotein fractions of blood plasma [25]. It was found that not all LDL circulating in human blood is atherogenic [25]; native LDL cholesterol does not cause lipid accumulation in the arterial wall [26]. In these ways, the presence of an LDL subfraction prone to multiple atherogenic modifications, including desialylation, the early and the most likely modification occurring in the blood of patients with atherosclerosis, was established [27,28,29,30].

Sialic acid is an important element of native LDL that represents the terminal carbohydrate of biantennary sugar chains in apolipoprotein B (apoB) and carbohydrate chains in gangliosides. Galactose, which is the monosaccharide residue preceding sialic acid in the carbohydrate chain, becomes terminal and exposed externally after the desialylation. This was used to isolate the desialylated LDL fraction from the total LDL using agglutinin *Ricinus communis* (RCA120), which has a high affinity to terminal galactose [31,32]. This method has allowed distinguishing between desialylated and sialylated LDL fractions of total LDL demonstrating the differences in multiple physicochemical parameters between desialylated and sialylated LDL [33]. In this regard, the study indicated that, compared to healthy people, the blood level of desialylated LDL was considerably higher (by 1.5- to 6-fold) and may account for up to 60% of total LDL in the blood of patients with coronary artery disease (CAD) [34]. Interestingly, a small fraction of desialylated LDL, approximately 5–10% of total LDL, can be identified in the blood of healthy people [27]. Moreover, desialylation is followed by a series of other physical and chemical LDL modifications, including particle size reduction, increase in its density and negative electrical charge, loss of lipids, and oxidation (accumulation of apoB-bound cholesterol) [19,28].

The sequence of multiple modifications of LDL particles was observed in the ex vivo experiments [35]. It was shown that desialylation of LDL occurred after 1 h of incubation of native LDL with serum obtained from atherosclerotic patients, and desialylated LDL was able to cause lipid retention after 3 h. Reduction in neutral lipids/phospholipids and particle size appeared after 6 h of incubation. LDL become more electronegative in 36 h. Finally, the loss of tocopherol, increasing susceptibility to copper oxidation, and accumulation of lipid peroxidation end products was detected within 48–72 h of incubation. In this way, properties of the multiply modified LDL (mmLDL) fraction were described. Thus, it was confirmed that desialylated LDL particles were smaller and denser than native LDL [33,36]. Correspondingly, the sdLDL subfraction displayed a profound deficiency in sialylation rate, compared to the native LDL and that clearly correlated with its increased atherogenicity [26]. Moreover, the desialylated LDL subfraction was more electronegative than native LDL [36]. Additionally, there was a direct correlation between LDL being electronegative (LDL(−)) and its desialylation rate suggesting that desialylated LDL and LDL(−) may belong to the same LDL subfraction [33,37]. In healthy subjects, the LDL(−) subfraction was shown to be highly enriched with desialylated LDL, which had a substantial reduction of sialic acid content, compared to native LDL [31,38,39,40]. Moreover, a negative correlation between the ability of LDL(−) and LDL cultured from uninvolved human aortic intima to induce intracellular lipid accumulation and the content of sialic acid in the lipoprotein particles was established [38]. These findings indicated that LDL(−) and aortic LDLs were low in sialic acid content, hence, they were considered to be desialylated lipoproteins. It is worth emphasizing that LDL oxidation, i.e., accumulation of cholesterol covalently bound to apoB, a marker of lipoperoxidation, occurs at the later stages in the chain of multiple modifications of LDL particle [35], pointing out that modified LDL has an increased susceptibility to oxidation in vitro [41]. At the same time, apoB lipoprotein was demonstrated to be glycated in sdLDL particles both in vitro and in vivo [42,43], and there was the inverse correlation between the level of glycated apoB and the particle size [44].

Remarkably, despite a large number of the experiments studying the role of oxidized LDL (oxLDL) in atherogenesis, artificially generated in vitro species of oxLDL remain undetectable in the blood. In fact, circulating mmLDL particles were found to exhibit signs of oxidation [41], however, LDL oxidation is likely to occur in the vascular wall but not in the blood. Whereas, numerous studies conducted during the past three decades indicated that other forms of atherogenic LDL modifications, such as LDL(−), sdLDL, desialylated LDL, and glycated LDL can be identified in the blood of atherosclerotic and diabetic patients [27,45,46,47,48,49]. In particular, LDL glycation occurs as a result of non-enzymatic reaction of glucose and its metabolites with free amino groups of apoB-100 lysine. As demonstrated in patients with metabolic syndrome and type II diabetes, sdLDL has high susceptibility to glycation [50,51]. Moreover, glycation makes LDL more sensitive to oxidation and formation of glycated LDL and other advanced glycation end products, which increases the atherogenic potential of LDL [50]. The enhanced atherogenicity of the sdLDL subfraction is related to its specific biochemical and biophysical features. The smaller-sized particles can easily penetrate into the arterial cells, where they serve as a source of cholesterol and subsequently lipid deposits. Due to the lower affinity for the LDL receptor, sdLDL particles have a prolonged time of circulation that increases the probability of their different atherogenic modifications in the blood plasma, such as oxidation, glycation, desialylation, and/or carbamylation. In addition, sdLDL also possesses high binding affinity to the proteoglycans contained in the intima layer of the arterial wall promoting subendothelial lipoprotein retention. Many reports indicated that elevated levels of sdLDL can be detected in several atherosclerosis-associated conditions, such as dyslipidemia, diabetes, and metabolic syndrome, as well as in some other disorders [51,52,53,54]. Regarding metabolic syndrome, high levels of sdLDL had an independent predictive value for subsequent cardiovascular events [55]. Additionally, oxidation of desialylated LDL increases the proatherogenic potential of LDL [56].

Thus, as compared to native LDL, desialylated LDL particles have a low content of sialic acid; they are smaller, denser, and more electronegative; they have increased lipid peroxidation; and contain more triglycerides and fatty acids [33]. Notably, desialylated LDL is different to native LDL in its carbohydrate, lipid, and apoB-100 structure [36]. Relying on the fact that all atherogenic LDLs described above have similar features, it is possible to suggest that modified LDLs represent the same lipoprotein particles subjected to multiple modifications. For example, the atherogenic LDL subfraction obtained from the blood of patients with diabetes corresponds with small-dense, glycated, and desialylated lipoprotein [47].

Furthermore, mmLDL is prone to a spontaneous aggregation and formation of complexes that further increases its atherogenicity. Thus, another LDL modification that is an essential condition for intracellular cholesterol accumulation is the formation of large highly atherogenic LDL-containing complexes, i.e., self-associates. It was noticed that without the formation of self-associates, even modified LDL does not cause the intracellular accumulation of lipids, hence it is not atherogenic [57]. The uptake of large LDL associates occurs through escaping the receptor-regulated pathway leading to excessive lipid accumulation in vascular cells. Several experiments showed that the presence of self-associates caused an increase in cholesterol ester content in cultured macrophages stimulating the formation of foam cells [58]. Furthermore, a positive correlation was observed between the atherogenic potency of modified LDL and a number of LDL self-associated complexes circulating in the blood of patients with CAD and diabetes mellitus [58]. In addition, the formation of LDL complexes with the components of connective tissue matrix, such as cellular debris, collagen, elastin, and proteoglycans is a further LDL modification that stimulates its uptake and the reduction of intracellular degradation of lipoproteins in these complexes leading to intracellular cholesterol retention [59,60,61].

In addition, LDL retained in the arterial wall can be also affected by enzymatic modifications since the following enzymes are hyperexpressed in the atherosclerotic plaque microenvironment: Cholesterol esterase (CEase), sphingomyelinase (SMase) or secretary phospholipase A2 (sPLA2) or proteases (matrix metalloproteases and cathepsins) [62]. The main atherogenic effect of enzymatic modifications is triggering LDL aggregation and fusion that support its subendothelial retention [63].

**Figure 1:**
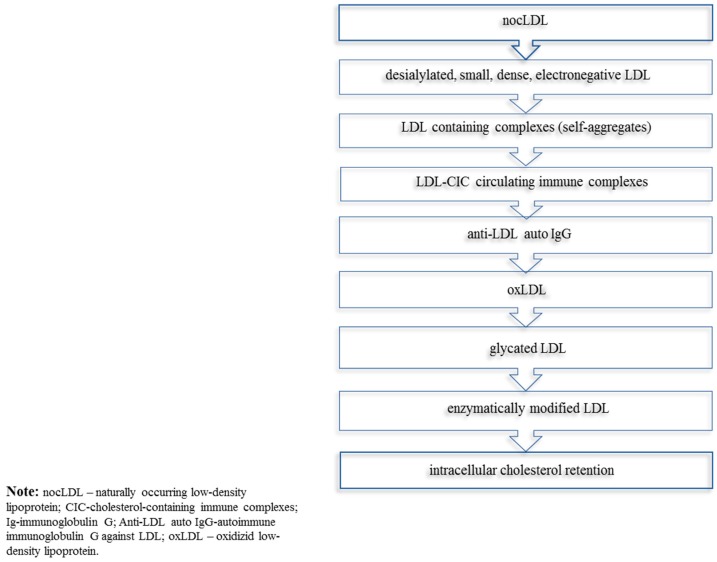
Multiply modified low-density lipoprotein (mmLDL) implicated in atherogenesis.

Relying on the above-described studies, it is possible to conclude that the circulating LDL of atherosclerotic patients sustains a cascade of consecutive modifications, such as desialylation, decrease in lipid content, reduction of the particle size, increase of its density and negative charge, lipid peroxidation, and the ability to aggregate that are indicative that it is atherogenic. Other non-enzymatic and enzymatic modifications can also affect circulating lipoproteins, therefore, also contributing to lipid accumulation in the arterial wall. It has to be stressed that desialylation is the most likely primary atherogenic modification of LDL, and hence, the most important modification in the initiation and development of atherogenesis. The subsequent series of LDL modifications are likely to represent a chain of multiple modifications that enhance its atherogenicity. The importance of LDL desialylation, in terms of inducing blood atherogenicity, will be described in the next subsection. The summary of multiple LDL modifications implicated in atherogenesis is presented in Figure 1.

### Desialylation of LDL Augments Its Atherogenicity

The accumulated evidence indicates that LDL desialylation is tightly implicated in the cholesterol retention process in the arterial wall. On comparison of the atherogenic and non-atherogenic features of LDL, the substantial difference in the sialic acid content of lipoprotein particles was observed [27]. There was a clear negative correlation between the LDL sialic acid content and the amount of cholesterol being accumulated in the vascular cells [33]. Other modifications, such as changes in LDL particle size, electrical charge, the content of phospholipids, neutral lipids, fat-soluble antioxidants, lipid peroxidation end products, and free lysine amino groups, as well as the extent of oxidation and oxidizability of LDL did not show any significant correlation with blood atherogenicity [33]. Moreover, compelling evidence has emerged recently indicating that desialylation is a key modification of LDL that occurs in the blood plasma of atherosclerotic patients. Using transcriptome analysis, 33 signaling pathways (SPs) accountable for the accumulation of cholesterol in macrophages cultivated in vitro with naturally occurring LDL were identified (our unpublished data) (Table 1). Thus, the latest experiments revealed that a complete majority (26 out of 33) of SPs attributed to naturally occurring LDL are also accountable for the desialylated LDL. As for oxidized LDL, it regulated only two SPs in the same way as naturally occurring LDL, and seven other SPs were regulated in the opposite way. Accordingly, it is possible to speculate that unlike LDL oxidation, LDL desialylation determines its atherogenic properties to a large extent. In addition, the atherogenicity of desialylated LDL, i.e., its ability to induce a substantial increase in intracellular cholesterol content, was proven by many laboratory experiments [39,64,65].

Two approaches were proposed to clarify the mechanisms of intracellular lipid accumulation triggered by desialylated LDL: The first, evaluation of binding, uptake, and degradation of LDL and the second, determination of hydrolysis and esterification rates of lipids in LDL particles. Desialylation was demonstrated to increase the sdLDL particle affinity to the proteoglycans in the arterial wall, therefore, enhancing its binding to the arterial proteoglycans and intracellular uptake, consequently increasing lipid accumulation in the arterial cells [66,67]. Moreover, binding to the scavenger receptor and the asialoglycoprotein receptor may account for the increased cellular binding and uptake of desialylated LDL [68]. Thus, it was established that the uptake of desialylated LDL was much higher than the uptake of native LDL, specifically by smooth muscle cells (SMCs) cultured from the atherosclerotic lesions, compared to the cells obtained from grossly normal aortic intima [68]. Conversely, the same study indicated that the rate of degradation of internalized desialylated LDL was lower than that of native LDL [68]. Hence, the accelerated uptake and the low rate of intracellular degradation of desialylated LDL can result in intracellular cholesterol accumulation. In addition, desialylated LDL stimulates intracellular esterification of free cholesterol via inhibition of the esterifying activity of cholesterol acyltransferase in macrophages, therefore, causing cholesterol accumulation [69].

Furthermore, it was shown that not only modified LDL was atherogenic but autoantibodies to modified LDL could also be responsible for blood atherogenicity [70]. The desialylated LDL subfraction is highly immunogenic since it can initiate the production of pro-atherogenic autoimmune immunoglobulins, predominantly immunoglobulin G (IgG). IgG may increase the LDL uptake by the aortic cells, and therefore, stimulate the formation of foam cells [71]. Considerably increased blood levels of circulating anti-LDL antibodies (IgG) were seen in atherosclerotic patients [70]. Noteworthily, the naturally circulating anti-LDL antibodies, such as IgM responsible for the specific recognition and clearance of modified LDL, and hence, playing an atheroprotective role were found in the blood of both atherosclerosis affected and apparently healthy people [71].

The formation of LDL cholesterol-containing circulating immune complexes (CICs) is also a sufficient condition for intracellular cholesterol retention. It was suggested that the exogenous anti-LDL antibodies are pro-atherogenic since their incubation with the normal human serum resulted in the formation of CICs and the stimulation of serum atherogenic properties [72]. Some studies demonstrated that mmLDL, as a part of CICs, possesses a higher atherogenic potential compared to the free modified lipoprotein and, therefore, is able to accelerate intracellular cholesterol retention [73,74]. Moreover, it was established that LDL isolated from the cholesterol-containing CICs represents mmLDL that has similar features to desialylated LDL: Small size, higher density, higher electronegative charge, lower content of sialic acid, increased oxysterol levels, and a similar amount of lipid peroxides [75,76]. LDL-containing CICs and anti-LDL auto-antibodies were detected in the blood of most patients with coronary atherosclerosis [76]. Moreover, a positive correlation between the levels of LDL-containing CICs and the severity of atherosclerosis was demonstrated [66]. In addition, LDL-containing immune complexes could be identified in both atherosclerotic plaques and the blood of apparently healthy children and newborns suggesting that proatherogenic changes in the blood may occur early in life [76]. These findings indicated that the desialylated LDLs have a significant atherogenic potency, and therefore, desialylation is the most important modification that determines the atherogenicity of lipoproteins.

## 3. The Role of Sialidases in Atherosclerosis

The enzyme trans-sialidase (neuraminidase), which belongs to the family of glycoside hydrolases (EC 3.2.1), was discovered to be accountable for the desialylation of the glycoconjugate of the lipoprotein particles, i.e., transferring sialic acid to different acceptors in the blood plasma [77]. This enzyme encompasses a broad substrate specificity by cleaving several sialosides, including α2-3, α2-6, and α2-8 [40]. Thus, LDL obtained from the blood of healthy donors incubated with neuraminidase, the structurally related enzyme to trans-sialidase, was partially desialylated [78]. Moreover, several experiments in vitro showed that desialylation of native LDL with trans-sialidase makes it capable of inducing accumulation of cholesterol in cells in the same way, as in patients affected with atherosclerosis [78,79]. Apart from human atherosclerotic plaques, trans-sialidase showed positive effects influencing atherosclerosis regression in the rabbit model of atherosclerosis [80]. The studies indicated that there is a difference between the activity and specificity of sialidases in atherosclerotic lesions and unaffected intima [81]. Additionally, it was found that the diminished trans-sialidase activity in the blood directly correlates with the lower ability of blood serum to induce cholesterol accumulation in a culture of unaffected human aortic intimal cells [82]. Indeed, the activity of trans-sialidase in human plasma determines blood atherogenic properties.

Furthermore, trans-sialidase can change the lipoprotein interaction with the arterial cells. It was shown that LDL desialylated by trans-sialidase causes intracellular accumulation of lipids, in association with stimulation of the proliferative activity of vascular cells and synthesis of extracellular matrix components [82]. Moreover, sialidases can change the activity of many blood cells and lipoproteins. The expression of hypomorphic sialidase in the blood cells of C57Bl/6 mice can alter lipoprotein metabolism, and that is a sufficient condition to attenuate atherogenesis [83]. Furthermore, strong evidence was presented supporting a central role of sialidases in the interaction between the uptake and production of lipoproteins. Downregulation of vascular LDL production modulated by hypomorphic sialidase expression reduced serum cholesterol levels in mice [83]. In addition, sialidases may be involved in the process of intimal thickening. In particular, Neu3 was recognized to be implicated in the growth, differentiation, and migration of vascular cells [84]. The overexpression of Neu3 inhibited matrix metalloproteinase-9 (MMP-9) expression in vascular SMCs, so this enzyme can modulate the vascular responses of SMCs and may participate in the destabilization of the atherosclerotic plaque [84]. Thus, relying on these observations, sialidases accountable for LDL desialylation, the principal modification that is responsible for its atherogenicity, play an important role in the atherogenesis and progression of atherosclerosis. Further studies of molecular mechanisms of trans-sialidase activity are required that would help to validate trans-sialidases as distinctive biomarkers for the diagnosis of subclinical and early atherosclerosis.

## 4. Diagnostic and Therapeutic Approaches

It is clear that lipidosis plays a crucial role in atherogenesis at the cellular and tissue levels. Therefore, it can represent a key target for the development of early intervention methods for diagnosis and anti-atherosclerotic therapy reducing the burden of CVD management and mortality. The discovery of the atherogenic modifications of lipoprotein particles in the blood of atherosclerotic patients has supported their good predictive and diagnostic value on the molecular level. It allowed identification of some specific prognostic and diagnostic methods for cardiovascular risk and subclinical and clinically manifested atherosclerosis (Table 2).

To date, none of the direct anti-atherosclerotic therapeutic methods is known. Therefore, a novel therapeutic approach, such as the use of trans-sialidase inhibitors was suggested [82]. Using trans-sialidase inhibitors may prevent LDL from desialylation and consequently from the formation of foam cells. It was demonstrated in apoE^−/−^ mice that administration of a sialidase inhibitor 2-deoxy-2,3-dihydro-*N*-acetylneuraminic acid (DANA) had an anti-atherogenic effect [82]. The activity of trans-sialidase in human blood plasma was also decreased with the use of some plant-derived extracts. In particular, the inhibitory effects of garlic seed extract (“Allicor” preparation) and pollen (“Pollinat” preparation) were described [89]. Furthermore, the cell-based (in vitro and ex vivo) models providing an alternative method for rapid screening of putative anti-atherosclerotic drugs while simultaneously allowing an estimation of the atherogenic potential of serum were explored [18,92,93]. The substances of natural origin tested on these cell models enabled the selection of preparations with high anti-atherogenic activity. Hitherto, three herbal preparations, namely Allicor, Inflaminat, and Karinat were developed and exhibited good tolerability and efficacy in clinical trials [89]. The availability of the cell-based test systems has facilitated a selection of novel natural agents with anti-atherogenic activity and provided the potential for the development of further herbal preparations for a long-standing anti-atherosclerotic therapy. Additionally, based on the use of immobilized LDL, a procedure for extracorporeal removal of anti-LDL antibodies (non-lipid atherogenicity factors) from circulation was developed [65]. Using LDL-apheresis, as a substitute technique, showed positive results [94]. These diagnostic and therapeutic approaches represent examples of the successful atherosclerosis management that can be adopted by clinical practice.

## 5. Conclusions

It was discovered that a subfraction of the circulating LDL of atherosclerotic patients sustains numerous alterations of protein, carbohydrate, and lipid moieties in the blood plasma, therefore, it was considered as mmLDL, and multiple modifications of LDL determine its atherogenicity. The modified LDL subfraction is capable of inducing the accumulation of lipids in the subendothelial cells, primarily cholesteryl esters that in turn is associated with other cellular atherosclerotic manifestations. Of note, the studies showed that LDL desialylation is the earliest modification, therefore, it plays the most prominent role in the process of cholesterol retention in the subendothelial cells. Relying on the molecular properties of mmLDL, novel specific diagnostic approaches were developed. There is a growing interest in non-pharmaceutical products of natural origin that can be explained by the toxicity and lower efficacy of synthetic substances. Therefore, the alternative therapeutic approaches, such as cell-based models were explored that may help to develop safer long-term treatment schemes using plant-derived compounds. To date, three herbal preparations, namely, Allicor, Inflaminat, and Karinat were developed, as effective anti-atherogenic remedies. Nonetheless, further studies based on the molecular mechanisms of lipid modifications, as well as high-quality large-scale human clinical trials are required, in order to build up a sufficient amount of evidence that would help to establish reliable prognostic/diagnostic biomarkers and therapeutic methods for subclinical and early atherosclerosis that can be applied in clinical practice.

## Figures and Tables

**Table 1:** Signaling pathways regulated by modified lipids causing lipid accumulation in macrophages.

Signaling Pathway	Lipid Modification		
	Naturally Occurring	Desialylation	Oxidation
EP2---> BDNF	↑	-	-
hypoxia pathways	↑	↑	-
insulin--->AKT-1 pathway	↑	-	-
Prostanoid receptor signaling	↑	↑	-
TBK1:TRIF:IKK-i--->p50:RelA	↑	↓	-
TNF-α--->p50:RelA-p65	↑	-	-
Akt-1---Mdm2--->AR	↓	↓	-
Caspase network	↓	-	-
cyclosome--->Nek2A	↓	↓	-
cyclosome---/SnoN	↓	↓	-
dsRNA--->c-Jun	↓	-	-
E1---PIRH2---/p53	↓	↓	-
Emi1---Cdc20---/cyclosome	↓	↓	-
Emi1---Fzr1---/cyclosome	↓	↓	-
ER-α pathway	↓	↓	↑
HMGCR regulation	↓	↓	-
Htt degradation	↓	↓	-
LT-betaR---NIK, RelB--->CCL19	↓	↓	↑
MAD2---Cdc20--->cyclosome	↓	↓	-
MEK--->ABP-280	↓	↓	-

Note: ↑—upregulated; ↓—downregulated.

**Table 2:** mmLDL-based specific prognostic/diagnostic methods of atherosclerosis.

Diagnostic Technique/Biomarker	References
Measuring total level of LDL-CIC	[75]
Detection of trans-sialidase activity	[82]
Measuring the proportion of mmLDL in serum	[85]
Measuring the levels of circulating oxLDL by a specific monoclonal antibody	[86,87]
Measuring the cholesterol content and apoB-100 in LDL-containing immune complexes	[88]
Simultaneous measurement of total and desialylated apoB-100 in serum and calculation of desialylated apoB-100 fraction size	[89]
Detection of the level of circulating anti-LDL antibodies (IgG and IgM)	[90,91]

Note: mmLDL—multiply modified low-density lipoprotein; oxLDL—oxidized low-density lipoprotein; apoB—apolipoprotein B; IgG—immunoglobulin G; IgM—immunoglobulin M; LDL-CIC—low-density lipoprotein containing circulating immune complexes.
